# Diagnostic approach, therapeutic strategies, and surgical indications in intradural thoracic disc herniation associated with CSF leak, intracranial hypotension, and CNS superficial siderosis

**DOI:** 10.1007/s10072-022-06059-y

**Published:** 2022-04-08

**Authors:** Giulio Bonomo, Alberto Cusin, Emanuele Rubiu, Guglielmo Iess, Roberta Bonomo, Giorgio Battista Boncoraglio, Mario Stanziano, Paolo Ferroli

**Affiliations:** 1 Department of Neurosurgery, Fondazione IRCCS Istituto Neurologico C. Besta, University of Milan, Via Giovanni Celoria 11, 20133 Milan, Italy; 2 University of Milan, Milan, Italy; 3 Department of Neurology, Fondazione IRCCS Istituto Neurologico C. Besta, Milan, Italy; 4 Experimental Neurology Unit, School of Medicine and Surgery, University of Milano-Bicocca, Monza, Italy; 5 Neuroradiology Unit, Diagnostic and Technology Department, Fondazione IRCCS Istituto Neurologico C. Besta, Milan, Italy; 6 Neurosciences Department “Rita Levi Montalcini, ” University of Turin, Turin, Italy

**Keywords:** Intradural thoracic disc herniation, CSF leak, Intracranial hypotension, CNS superficial siderosis

## Abstract

**Background and purpose:**

Intradural disc herniation (IDH) can manifest with radicular or medullary syndrome. In about 15% of cases, IDH may be responsible, through a dural laceration, for a CSF leak, determining spontaneous intracranial hypotension (SIH) and CNS superficial siderosis (CNSss). This paper attempts to present an overview on IDH as the cause for both CSF leak, and subsequent SIH, and CNSss, and to describe a peculiar clinical and neuroradiological scenario related to this condition.

**Methods:**

A search on the PUBMED database was performed. Although the investigation did not rigorously follow the criteria for a systematic review (we consider only articles about thoracic IDH), nonetheless, the best quality evidence was pursued. Furthermore, an illustrative case was presented.

**Results:**

A 69-year-old woman was referred to our hospital for slowly progressive gait disturbances and hearing impairment. Brain imaging revealed diffuse bilateral supratentorial and infratentorial superficial siderosis, mostly of the cerebellum, the eighth cranial nerves, and the brainstem. Spinal imaging disclosed a posterior disc herniation determining a dural tear at D6-D7. Lumbar puncture revealed low opening pressure and hemorrhagic CSF with siderophages. A posterior transdural herniectomy and dural sealing determined a stabilization of hearing and a significant improvement in both gait and balance.

**Conclusions:**

The diagnostic workup of CNSss with suspected CNS leak demands whole neuraxis imaging, especially in cases presenting SIH or myelopathic symptoms. This may avoid delays in detection of IDH and spinal dural leaks. The different forms of treatment available depend on the type and severity of the clinical picture.

## Introduction

Intradural disc herniation (IDH) consists of a nucleus pulposus fragment of the intervertebral disc violating the dural sheath and penetrating the thecal space. The first report of an IDH was made by Dandy in 1942 [1]. IDH has an incidence rate of about 0.27 to 0.33% of all cases of disc herniation, and its incidence peaks in the fifth and sixth decades of life [2]. Approximately, 92% of IDHs occur in the lumbar region, most commonly in the L4-L5 segment (55%), followed by L3-L4 segment (16%), and L5-S1 segment (10%) [3]. By contrast, less than 5% occur in either the cervical or thoracic segments [4]. The main clinical sign of thoracic IDH is represented by radicular pain [4]. Thoracic IDH may also manifest with walking ataxia or pyramidal tract syndrome, raising the suspect of medullary compression. Other less common signs include axial pain (cervical above T5 level and lumbar below T10 level), scapulalgia for T1–T2 locations, radiating pain in the T1 territory, and Bernard Horner’s syndrome due to T1 root’s sympathetic component compression. IDH may also result in a dural laceration, which, in turn, can promote a CSF leak, whose incidence has been reported to be up to 15% [5]. CSF leak may be easily responsible for spontaneous intracranial hypotension (SIH) and CNS superficial siderosis (CNSss). While SIH mainly manifests primarily with headache, CNSss becomes explicit with hearing and gait impairments. Both SIH and CNSss are also characterized by typical radiological findings.

In this paper, we review the literature concerning thoracic IDH as the cause for both CSF leak, and subsequent SIH, and CNSss. We also present a case exposing the peculiar clinical picture, discussing the possible underlying pathophysiological mechanisms and examining the therapeutic strategies that were implemented.

## Materials and methods

Following a brief overview about the rationale, indications, pathophysiology, and results of the implemented surgical treatments, we will discuss the implications according to the available literature. With this aim, we performed a search on the PUBMED database for the following: thoracic intradural disc herniation, spontaneous intracranial hypotension, central nervous system superficial siderosis, and cerebrospinal fluid leak. The search was conducted on the available literature before December 2021, comprising only articles with full text in English, without any historical limitation. The literature search did not strictly follow the criteria for a systematic review because we consider only articles about thoracic intradural disc herniation. Nevertheless, we tried to identify the highest quality of available evidence for each technique implemented in the case of thoracic intradural disc herniation. Considering the extreme heterogeneity and the limited number of articles, we present the data as a comprehensive and narrative review. Moreover, we have included the experience of our Institution with an illustrative case. All the reported techniques are available and used at our Institution. All MRI and CT scan images included in this review were evaluated by members of the Neuroradiology Unit. IDH, SIH, and CNSss were diagnosed according to pre-specified clinical and radiological criteria.

## Case presentation

**Fig. 1:**
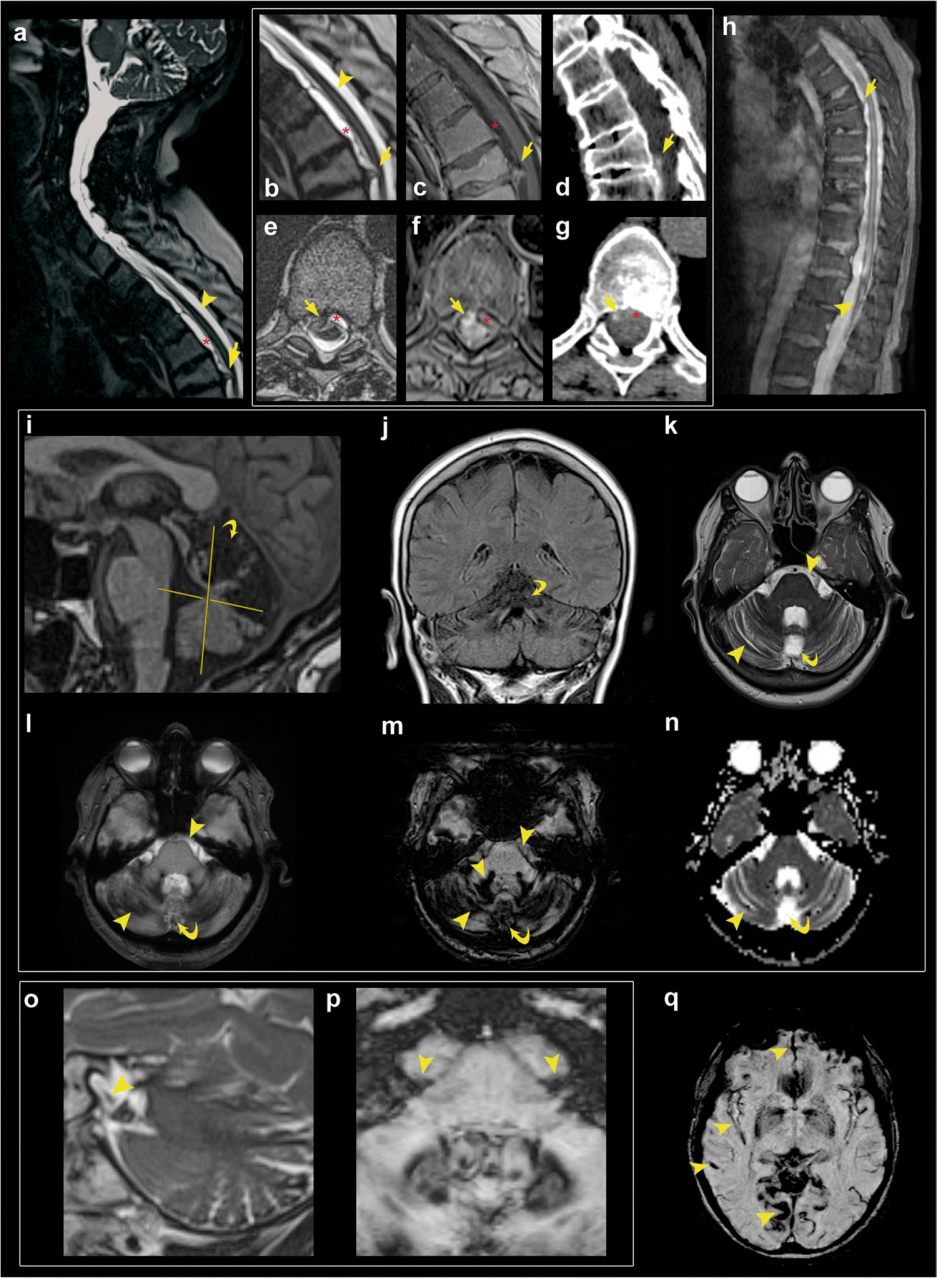
Non-contrast sagittal Constructive Interference in Steady State (CISS) (**a**), Turbo-Spin Echo (TSE) T2 (**b**), and T1 (**c**) Magnetic Resonance (MR) images of the cervical and upper thoracic spine show an anterior wedge degenerative deformation of the D6 vertebral body with superimposed a prominent central disc extrusion characterized by osteo-calcific signal (yellow arrow) determining a dural tear at D6-D7 level. Above the disc extrusion, note the “sentinel” epidural fluid collection (red asterisk) stretching along the ventral aspect of the spinal canal and displacing the dura posteriorly. The partially calcified disc extrusion is also recognizable by non-contrast sagittal spinal Computed Tomography (CT) images (**d**). Axial T2 (**e**) and T1 (**f**) TSE as well as CT (**g**) images through the D6–D7 disc clearly show a dural defect just on the right of the midline at the level of the spur and further delineate the associated ventral epidural fluid collection. CISS (**a**) and TSE T2 (**b, e**) MR images show low signal intensity along the surface of the spinal cord consistent with superficial siderosis (SS, yellow arrowhead); extensive SS around the spinal cord, also below the dural defect and up to the medullary cone, is best demonstrated by Sagittal Gradient-Echo (GRE) T2 MR images of the thoracic spine (**h**). Non-contrast sagittal 3D T1 (**i**), coronal T2 Fluid-attenuated inversion recovery (FLAIR) (**j**), axial TSE T2 (**k**), GRE (**l**), susceptibility weighted (SW) (**m**), and apparent diffusion coefficient (ADC) (**n**) images show a specific pattern of superior cerebellar atrophy (yellow crooked arrow) associated with infratentorial diffuse SS (yellow arrowhead), mostly of the pons, cerebellar folia, dentate hila, and superior vermis. SS along the eighth cranial nerves on both sides is clearly seen by Sagittal TSE T2 (**o**) and axial GRE (**p**) magnification. Supratentorial SS (yellow arrowhead) along the interhemispheric and Sylvian fissures, as well as within temporal and occipital sulci is well demonstrated by the axial susceptibility weighted (SW) sequence (**q**)

**Fig. 2:**
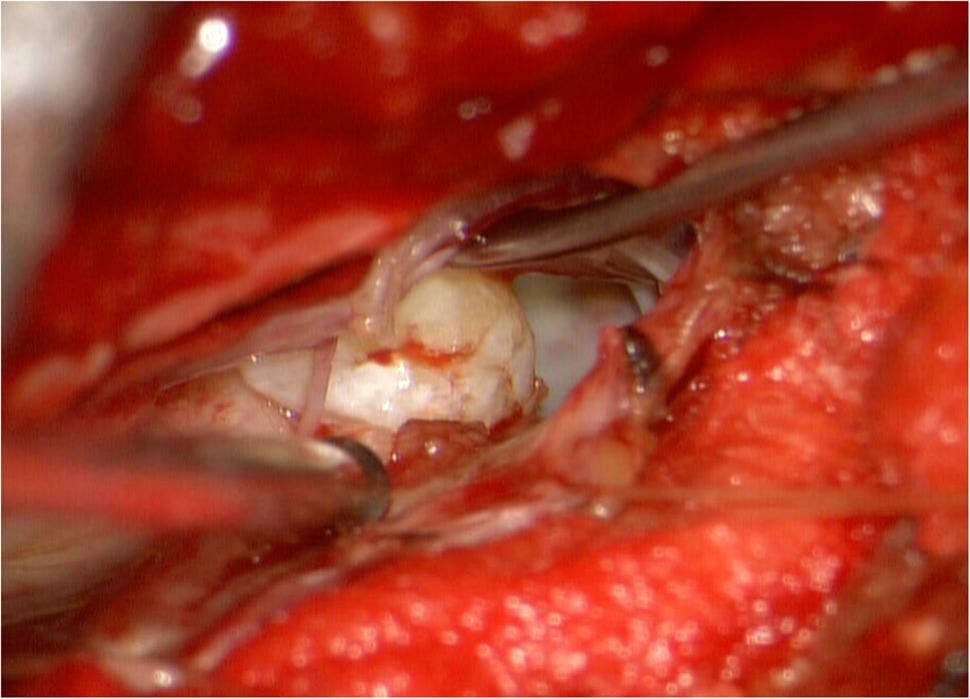
Intraoperative picture illustrating the ventral intradural disc herniation after posterior durotomy

We present the case of a 69-year-old, right-handed, woman who suffered from a 1-year history of slowly progressive gait disturbances and hearing impairment. Neurological examination revealed an ataxic gait and bilateral sensorineural auditory alteration. No previous history of surgery or trauma of the CNS were present, and no major comorbidities were reported. Therefore, the patient underwent brain magnetic resonance imaging (MRI) (Fig. 1) which disclosed diffuse bilateral supratentorial and infratentorial superficial siderosis, mostly of the cerebellum, the eighth cranial nerves, and the brainstem. Hemosiderin coating was specifically accentuated at the level of the cerebellar folia. This appearance was combined with a prominent and symmetrical secondary degenerative atrophy of the vermis and superior cerebellar peduncles. No apparent signs of SIH were present. Spinal MRI and computed tomography (CT) (Fig. 1) showed an anterior dural detachment with a ventral epidural fluid collection extending from D6-D7 up to D1. There was also an osteoporotic fracture with anterior wedge deformation of the D6 and D7 vertebral bodies with superimposed posterior marginal-somatic osteophytosis and herniation of disc material with osteocalcific signal that determined a dural tear at D6-D7. Moreover, fibrotic reactive tissue was recognizable at the level of dural defect. Lumbar puncture showed a low opening pressure (3 cm H2O) and hemorrhagic CSF with 40/mm3 red blood cells and siderophages. In October 2021, the case was discussed by a multidisciplinary committee which set the indication for surgical repair of the dural fistula. The patient gave written informed consent for the procedure. The day of surgery, the patient was positioned prone and, under microscopic guidance, a D6-D7 herniectomy through a right hemilaminectomy combined with an intradural approach was performed. (Fig. 2) A muscle patch and fibrin glue were used to seal the ventral dural defect. Histology confirmed degenerative disc material. Postoperative course was uneventful and characterized by early autonomous mobilization. The patient was discharged from our Institute 2 days after the surgical procedure. She exhibited no signs or symptoms of intracranial hypotension at the time of the discharge and at 3 months follow-up. In addition, she reported a stabilization of the hearing impairment but also a significant improvement in both gait and balance in the following 3 months after surgery.

## Literature review

In 2014, Toro et al. described the case of a patient with a 4-year history of leg weakness and progressive gait impairment, besides other symptoms such as bilateral hearing loss and urinary incontinence. T2-weighted brain MRI showed extensive hemosiderin deposits around the brainstem and along the cerebellar folia, while spinal MRI demonstrated a disc herniation at the T8-T9 level, located where a previous dynamic CT myelogram highlighted a ventral dural defect. A surgical dural repair was offered but declined by the patient [6]. Reviewing this case, we believe that in cases of thoracic IDH with progressive myelopathy and/or CNSss, a more invasive treatment is needed through a total microdiscectomy.

In 2018, Wipplinger et al. described a case of CNSss secondary to a thoracic IDH causing CSF leak. Their patient was successfully treated with lateral T6-T8 transpedicular partial corpectomy, as well as diskectomy with decompression and fusion, followed by watertight closure of the CSF leak. No postoperative complications occurred, and, at 3 months’ follow-up, the patient displayed stability of preoperative symptoms, such as mild bilateral hypoacusis and mild difficulty in tandem gait [7].

In 2020, Cornips et al. presented two patients with a thoracic IDH, secondary SIH and, in one of them, CNSss [8]. The first patient, without CNSss, presented with frontal headache, mild cognitive disfunction, and gait impairment. Cranial MRI showed bilateral subdural effusions and sagging of the midbrain. On the suspicion of a spinal CSF leak, MRI of the entire neuraxis was also performed, demonstrating a left paramedian thoracic IDH at the T9-T10 level. After refractoriness to both thoracic and lumbar EBP, a left-sided thoracoscopic microdiscectomy was performed, resulting in clinical improvement [8]. The second patient, with CNSss, presented with a 2-year history of pain in the occipital region. Cranial MRI indicated subarachnoid susceptibility artifacts, particularly in the posterior fossa. As both history and MR angiography were not indicative for subarachnoid bleeding, a diagnosis of CNSss was formulated. Spinal MRI revealed a large central thoracic IDH at the T7-T8 level. Hence, a left-sided tubular microscopic discectomy was performed. A few days after surgery, the patient became dyspneic and a CT-thorax demonstrated a large fluid collection in the left hemithorax, thus posing an indication for an external pleural drainage, which allowed significant improvement of the clinical picture [8]. In our opinion, in the presence of a thoracic IDH with symptomatic SIH and CNSss, the conservative EBP does not represent the most effective therapeutic strategy. At the same time, in the presence of mild SIH and/or CNSss symptoms, a thoracoscopic approach for microdiscectomy represents a far too invasive procedure.

## Discussion

The migration of a disc into the intradural region requires perforation of the annulus fibrosus, and then the laceration of both the posterior longitudinal ligament and the dura mater. Various predisposing anatomical factors have been proposed, of which the most relevant one is the presence of adhesions between the annulus fibrosus, the posterior longitudinal ligament, and the dura mater. These adhesions are likely to be congenitally present or occur secondary to chronic disc protrusion [4]. In this regard, over the long term, a herniated disc does not represent an inert tissue, but rather an active area of proliferation, neovascularisation, and inflammation. The inflammatory response (expressed by the production of proinflammatory cytokines) leads to calcification of the disc, which, in turn, causes intradural erosion, and subsequently, tears [9]. Lesions of the dura mater, in turn, may lead to SIH [10]. CSF leak is also associated with CNSss [11, 12], a rare disorder characterized by the deposition of hemosiderin in the leptomeninges and the subpial layer of the cerebellum, VIII cranial nerve, brainstem, and spinal cord, resulting in severe neurological deficits, such as ataxia and hearing impairment [13].

MRI yields pathognomonic findings, which may be revealed in the presymptomatic phase of the disease. They result from the deposits of hemosiderin, ferritin, and ionic iron. In gradient-echo T2-weighted images, the hallmark is a dark band surrounding the intracranial structures, such as the cerebellar hemispheres and the VIII cranial nerve, while other cranial nerves are less frequently affected. On the other hand, CT imaging is not sufficient for the diagnosis of CNSss [14].

The main differential diagnosis of CNSss are neoplasms (21%), cranial or spine trauma (13%), vascular malformations (9%), neurosurgical procedures (7%), and cerebral amyloid angiopathy (3%). Nevertheless, most cases remain idiopathic (35%) [15].

The association between CSF leak and CNSss has been explained by two theories. The first theory states that brain sagging due to intracranial hypotension may lead to bleeding from bridging veins on the surface of the cerebellum [16]. While the second theory affirms that the bleeding source may be localized around the dural defect [12, 17, 18]. In fact, regarding the latter theory, when CSF leaks through a dural defect, the epidural space enlarges, stretching the epidural venous plexus and making it more prone to laceration. Therefore, when bleeding from this plexus occurs, it may not stop spontaneously because of CSF’s continuous flow. This, in turn, can lead to subarachnoid bleeding.

Preoperative imaging-based diagnosis of thoracic IDH is challenging. Considering the literature [19–29], in only a few cases the presence of thoracic IDH was suspected following preoperative investigation with CT myelography. In our case, preoperative diagnosis through spine MRI was possible by understanding the underlying pathophysiological mechanism that binds CNSss, SIH, and IDH. However, when preoperative imaging-based diagnosis of IDH is non-discerning, an alternative route must be undertaken and therefore diagnostic workup for SIH should be implemented. SIH can be identified through neuroradiological exams such as brain MRI. Through brain MRI, five typical features can be identified: subdural fluid collections (50%), diffuse non-nodular enhancement of the pachymeninges (due to dilated blood vessels in the subdural area), engorgement of venous structures, pituitary hyperemia, to the point of mimicking a pituitary tumor [30] and sagging of the brain [31]. Both the dural enhancement and pituitary enlargement are consistent with the Monro–Kellie doctrine: in the presence of a significant decline in CSF flow, intracranial blood volume must increase to keep the total intracranial volume constant. In line with this theory, subdural hematomas could be the expression of an underlying CSF leakage and they usually can be managed by treating the spinal CSF leak [32]. In addition to MRI imaging, brain CT scan can suggest the diagnosis by highlighting subdural fluid collections or obliteration of subarachnoid cisterns [33]. Even myelography with iodinated contrast followed by spine CT can accurately define the location of a CSF leak [34].

Surgical treatment of thoracic IDH is needed in the presence of radicular symptoms, myelopathy clinical and radiological signs (highlighted by a medullary T2 hyperintensity area on spine MRI), and symptomatic SIH [35]. In our case, symptoms were ascribed to CNSss, thus posing a new and rare surgical indication in the case of an IDH. Three types of surgical approaches are now used in the treatment of thoracic IDH: (1) posterolateral with pedicular-transfacet and transfacet variations that spare the pedicle; (2) lateral such as costotransversectomy; and (3) anterior such as transpleural thoracotomy, thoracoscopy, and mini-thoracotomy. The choice of approach depends on patient’s characteristics (weight) and on the location (central or lateral), size, and type (soft or calcified) of herniation. On the other hand, treatment of SIH begins with conservative management, including bed rest, intravenous administration of fluids and steroids. If the patient fails to respond to medical therapy, epidural blood patch (EBP) is adopted [36], either by percutaneous or open surgical approaches. The mechanism of action of EBP is based on its initial tamponade effect over the dural tear and subsequent scar formation [37].

In our case, the clinical picture was mainly ascribed to CNSss, which, undoubtedly, set the indication to invasive treatment as to resolve the CSF fistula. Given the absence of myelopathy and significant SIH, our surgical strategy was primarily based solely on repairing the ventral dural defect to handle the progression of frank CNSss. Therefore, we proceeded with a posterior transdural herniectomy through a monolateral laminectomy and the placement of muscle and fibrin glue to repair the anterior dural fistula, rather than performing a more invasive total microdiscectomy.

## Conclusions

In the case of clinal and radiological signs of CNSss and/or SIH associated with suspected CSF leak, diagnostic workup should include MRI of the whole spine, especially in patients with myelopathic symptoms that could be ascribed to IDH. This may avoid delays in detection and treatment of spinal dural CSF leaks. With no apparent myelopathy, a less demolitive surgery is recommended for IDH, proceeding with the repair of the fistula with sealants and the excision of the intradural herniated disc material.

## Data Availability

The data that support the findings of this study are available from the corresponding author upon reasonable request.

## Abbreviations

ADC: Apparent diffusion coefficient
CISS: Constructive interference in steady state
CNS: Central nervous system
CNSss: CNS superficial siderosis
CSF: Cerebrospinal fluid
CT: Computed tomography
EBP: Epidural blood patch
FLAIR: Fluid-attenuated inversion recovery
GRE: Gradient-echo
IDH: Intradural disc herniation
MR: Magnetic resonance
MRI: Magnetic resonance imaging
SIH: Spontaneous intracranial hypotension
SS: Superficial siderosis
SW: Susceptibility weighted
TSE: Turbo-spin echo
